# Application of a roller conveyor type plasma disinfection device with fungus-contaminated citrus fruits

**DOI:** 10.1186/s13568-020-01177-2

**Published:** 2021-01-09

**Authors:** Akikazu Sakudo, Yoshihito Yagyu

**Affiliations:** 1 School of Veterinary Medicine, Okayama University of Science, Imabari, Ehime Japan; 2 Laboratory of Biometabolic Chemistry, School of Health Sciences, University of the Ryukyus, Nishihara, Okinawa Japan; 3 Department of Electrical and Electric Engineering, Sasebo National College of Technology, Sasebo, Nagasaki 857-1193 Japan

**Keywords:** *Citrus*, Disinfection, Dielectric barrier discharge, Fruits, Fungi, Plasma

## Abstract

Efficient methods to achieve the safe decontamination of agricultural products are needed. Here, we investigated the decontamination of citrus fruits to test the antifungal potential of a novel non-thermal gas plasma apparatus, termed a roller conveyer plasma instrument. This instrument generates an atmospheric pressure dielectric barrier discharge (APDBP) plasma on a set of rollers. *Penicillium venetum* was spotted onto the surface of the fruit or pericarps, as well as an aluminium plate to act as a control, before performing the plasma treatment. The results showed that viable cell number of *P. venetum* decreased with a decimal reduction time (*D* value or estimated treatment time required to reduce viable cell number by 90%) of 0.967 min on the aluminium plate, 2.90 min and 1.88 min on the pericarps of ‘Kiyomi’ (*Citrus unshiu* × *C. sinensis*) and ‘Kawano-natsudaidai’ (*C. natsudaidai*) respectively, and 2.42 min on the surface of ‘Unshu-mikan’ (*C. unshiu*). These findings confirmed a fungicidal effect of the plasma not only on an abiotic surface (aluminium plate) but also on a biotic surface (citrus fruit). Further development of the instrument by combining sorting systems with the plasma device promises an efficient means of disinfecting citrus fruits during food processing.

## Introduction

Measures to control plant pathogens in primary food production are important for food quality management (Strange and Scott 2005). Losses caused by pathogens, animals and weeds are estimated to reduce global food productivity by 20–40% (Savary et al. 2012). According to reports from the Ministry of Agriculture, Forestry and Fisheries of Japan in 2019, 78,300 tons of citrus fruit was lost due to these issues from a total of 746,700 tons (Ministry of Agriculture, Forestry and Fisheries 2020), which cost 15,660 million yen (provisional price: 200 yen/kg). One of the major causes for these losses is fungal infection that occurs during the storage of citrus fruit after harvest.

Fungi are one of the major risk factors for plant diseases. For decades pesticides have been used to treat citrus fruit, but these chemicals are now known to pose a threat to human and animal health (WHO and FAO 1996; FAO and WHO 2016). Furthermore, efficient transportation systems for agricultural products have accelerated the spread of fungal diseases. As a consequence, these plant pathogens have a major environmental and economic impact because they can greatly reduce crop production. Therefore, an efficient and safe technology to disinfect foods during food processing is required. These considerations prompted us to develop an instrument that could be used to eradicate fungi from agricultural products.

Thermal disinfection technologies sometimes reduce the quality and nutritional value of foods (Huang et al. 2009; Wang et al. 2013). As a result, fresh produce such as fruits are usually disinfected with a solution of chlorine. However, residual chlorine can have a carcinogenic effect on humans and therefore represents a potential health risk (Rico et al. 2007; Allende et al. 2008; Chen and Hung 2017). Several alternative non-thermal disinfection technologies such as radiation (Molnár et al. 2018), hydrostatic pressure (Wang et al. 2016), ozone (Venta et al. 2010), electrolyzed water (Rahman et al. 2016), electrically charged disinfectant (Nakashima et al. 2017; Sakudo et al. 2020a, b), electroporation (Uemura and Isobe 2002), high power ultrasound (Bermudez-Aguirre 2017), high pressure (Ogawa et al. 2000), pulsed electric field (Garner 2019) and ultraviolet light (Manzocco et al. 2011), have been used to disinfect fruit. Unfortunately, some of these technologies give disappointing levels of disinfection or require excessive setup costs with specially trained personnel (Sakudo and Shintani 2010).

Plasma is often referred to as the fourth state of matter. Treatment with plasma is a promising non-thermal technique for decontaminating food products and represents a potential alternative to conventional disinfection methods (Afshari and Hosseini 2014; Ito et al. 2018; Sakudo et al. 2019; Charoux et al. 2020; Braný et al. 2020). A variety of bacteria and viruses as well as fungi have been shown to be inactivated by gas plasma treatment (Sakudo and Shintani 2010; Afshari and Hosseini 2014; Shintani and Sakudo 2016; Bermudez-Aguirre 2019). Recently, we developed a plasma device, known as a roller conveyer plasma instrument, which generates plasma via an atmospheric pressure dielectric barrier discharge (APDBD) (Toyokawa et al. 2017, 2018). By connecting to a wire motor, the roller conveyer plasma instrument can disinfect crops on rollers during the sorting process. Thus, the roller conveyer plasma instrument is a promising means of efficiently disinfecting crops. However, to date, the disinfection potential of this instrument for fruits contaminated with fungus remains unclear.

Here, we investigated the disinfection efficiency of the roller conveyer plasma instrument using citrus fruits contaminated with a fungus.

## Materials and methods

### Roller conveyer plasma instrument

We used a roller conveyer plasma instrument, which was previously reported (Toyokawa et al. 2017, 2018). The gas plasma apparatus comprises rolling electrodes and a high voltage power supply. The electrode is made up of a plastic rod (30 mm diameter) covered with a thin aluminium and silicon sheet (0.5 mm thickness), which was placed at an interval of 50 mm between a high voltage electrode and an earth electrode. The high voltage electrode was connected to an alternating power supply [V_pp_ (peak-to-peak voltage) = 10 kV; Frequency = 9–11 kHz; LHV-10AC, Logy Electric Co., Ltd., Tokyo, Japan]. The voltage waveforms in the steady condition (without any object placed on the electrodes) and in the object treatment condition (with an aluminium plate or citrus fruit) showed V_pp_ = 11.87 kV (maximal peak = + 6.56 kV; minimal peak = − 5.31 kV) and Frequency = 8.85 kHz in both cases (Additional file 1: Figure S1). The apparatus generated APDBD plasma in air.

### Fungal cultures and APDBD treatment

*Penicillium venetum* [American Type Culture Collection (ATCC) 16025] and *Aspergillus brasiliensis* (ATCC 16404) were used as test organisms in this study. The colonies that appeared after culturing the fungi on Potato Dextrose Agar (PDA) medium (FUJIFILM Wako Pure Chemical Corporation, Osaka, Japan) for 2 weeks were suspended in 1 ml of sterilized distilled water (Otsuka Pharmaceutical Co., Ltd., Tokyo, Japan). A 20 μl aliquot of fungal suspension was spotted onto either an aluminium plate (0.3 mm thickness), the pericarps of ‘Kiyomi’ (*C. unshiu* × *C. sinensis*) and ‘Kawano-natsudaidai’ (*C. natsudaidai*), or the surface of ‘Unshu-mikan’ (*C. unshiu*). The different samples were then subjected to APDBD treatment. Before treatment, the spots were air-dried and then set onto the grounding position. Plasma was generated at the grounding position between the silicon sheet and either the aluminium plate or the pericarp/surface of the citrus fruit. The plasma-treated fungal samples were suspended in sterilized distilled water (Otsuka Pharmaceutical Co., Ltd.) and the number of colony-forming units (CFU) per ml measured by calculating the colony number of the fungi on PDA plate medium after incubation at 25 °C for 3 days.

### Determination of decimal reduction time (*D* value)

*D* value was defined as the treatment time required for decreasing the original number of viable cells by 90%. The following equation was employed for this calculation:

$$ D = { 1}/\left( {\Delta {\text{logN}}/\Delta {\text{t}}} \right), $$

where ∆t is the time for a one log_10_ reduction in viable cells and ∆logN is the logarithmic value of the changes in the CFU/ml of fungi after APDBD treatment (Sakudo and Shintani 2010).

### Real-time polymerase chain reaction (PCR)

The APDBD-treated spots were taken up in distilled water (Otsuka Pharmaceutical Co. Ltd.) and boiled for 15 min to extract the genomic DNA. The resultant samples were subjected to real-time PCR analysis to quantify intact genomic DNA using Takara SYBR Premix Ex TaqTM II (Tli RNaseH Plus, Takara Bio Inc.) and specific primers for *Penicillium* 28S rDNA D1/D2 region (target size: 107 bp), including Forward primer (5′-GGG ACG TCA TAG AGG GTG AG-3′) and Reverse primer (5′-CCA CCC ATT TAG AGC TGC AT-3′), as well as for *Aspergillus* 28S rDNA D1/D2 region (target size:159 bp), including Forward primer (5′-CGG AGT CCG CAT TGT AAT TT-3′) and Reverse primer (5′-AGC TGC ATT CCC AAA CAA CT-3′). Amplification conditions were as follows. After heating at 95˚C for 30 s, the fluorescence intensity of the samples was measured during the PCR comprising 40 cycles of 95˚C for 5 s and 60˚C for 30 s using a Thermal Cycler Dice Real-time System (Takara Bio Inc.). The amplified DNA was verified by dissociation curve analysis. The PCR product was sized by agarose gel electrophoresis.

### Statistical analysis

The results are shown as the mean ± SEM (standard error of the mean) of experiments, which were performed in triplicate. Non-repeated analysis of variance (ANOVA) followed by Bonferroni correction was employed to the statistical analysis of significant difference using the GraphPad Prism 7.02 software (GraphPad Prism Software Inc., La Jolla, CA).

## Results

### Decrease in the viable cell number of *P. venetum* spotted onto an aluminium plate after APDBD treatment

**Fig. 1:**
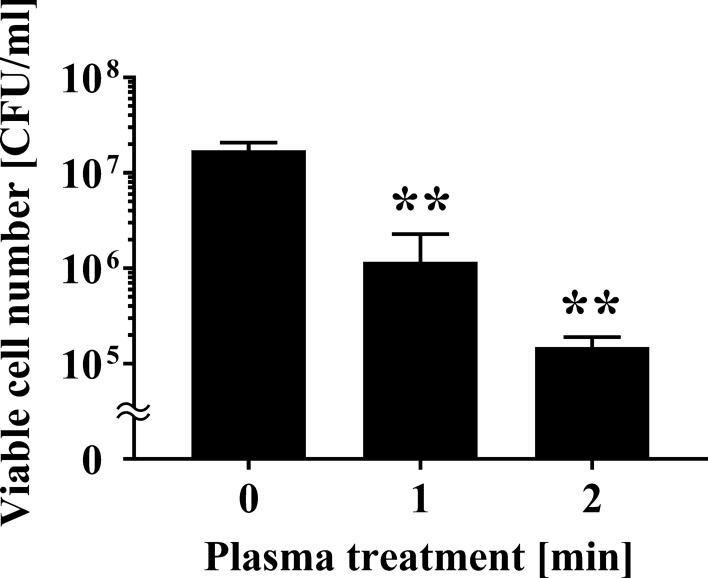
Viable cell number of *P. venetum* on an aluminium plate following APDBD treatment. The viable cell number of *P. venetum* on the contaminated aluminium plate at each individual APDBD treatment time (0, 1, or 2 min) is expressed as colony forming units (CFU) per ml. Differences where *p* < 0.01(**) versus control (0 min) were considered significant when verified by the non-repeated measured ANOVA followed by the Bonferroni correction

We investigated the changes in the viable cell number after APDBD treatment of *P. venetum* using a roller conveyor plasma instrument. Initially, a 20 μl aliquot of fungal suspension was spotted onto an aluminium plate, air-dried, and treated with APDBD for 0, 1, or 2 min. Viable cell counts were then performed. The results of viable cell numbers were 1.73 × 10^7^ ± 0.20 × 10^7^ CFU/ml at 0 min, but significantly decreased to 1.17 × 10^6^ ± 0.65 × 10^6^ CFU/ml at 1 min and 1.50 × 10^5^ ± 0.23 × 10^5^ CFU/ml at 2 min treatment time (Fig. 1). From the data, *D* value (decimal reduction time), which is the estimated treatment time required to reduce the number of viable microorganisms by 90%, was 0.967 min.

### Decrease of viable cell number of *A. brasiliensis* after APDBD treatment

**Fig. 2:**
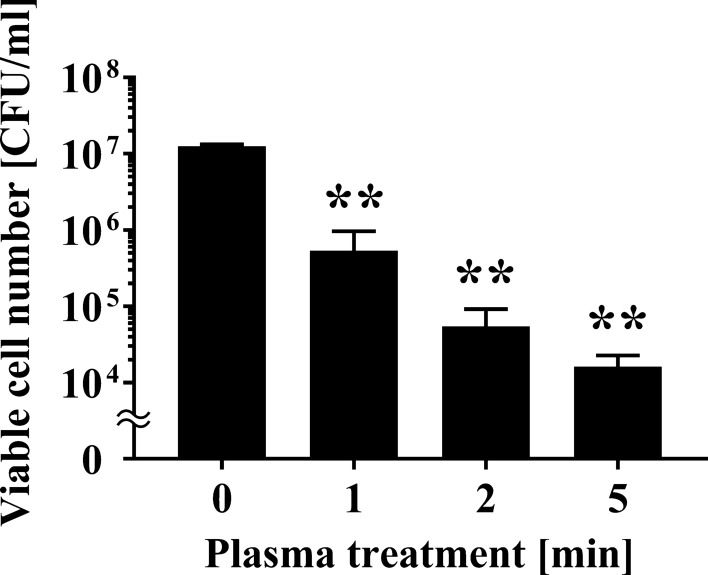
Viable cell number of *Aspergillus brasiliensis* spotted onto an aluminium plate following APDBD treatment. Viable cell number of *A. brasiliensis* spotted onto an aluminium plate, expressed as CFU/ml, after different APDBD treatment times (0, 1, 2, or 5 min). Differences where *p* < 0.01(**) versus Control (0 min) were considered significant when verified by the non-repeated measured ANOVA followed by the Bonferroni correction

The change in the viable cell number of *A. brasiliensis* after plasma treatment was investigated using a roller conveyor type plasma instrument. A 20 μl aliquot of fungal suspension was spotted onto an aluminium plate, air-dried, and treated with APDBD for 0, 1, 2, or 5 min. Viable cell number at 0 min was 1.25 × 10^7^ ± 0.04 × 10^7^ CFU/ml, whereas the APDBD treatment significantly decreased viable count to 5.37 × 10^5^ ± 2.48 × 10^5^ CFU/ml at 1 min, 5.47 × 10^4^ ± 2.17 × 10^4^ CFU/ml at 2 min, and 1.62 × 10^4^ ± 0.38 × 10^4^ CFU/ml at 5 min (Fig. 2). Based on the data from 0 to 2 min, the *D* value was 0.826 min.

### The effect of plasma treatment on DNA of *P. venetum* and *A. brasiliensis*

**Fig. 3:**
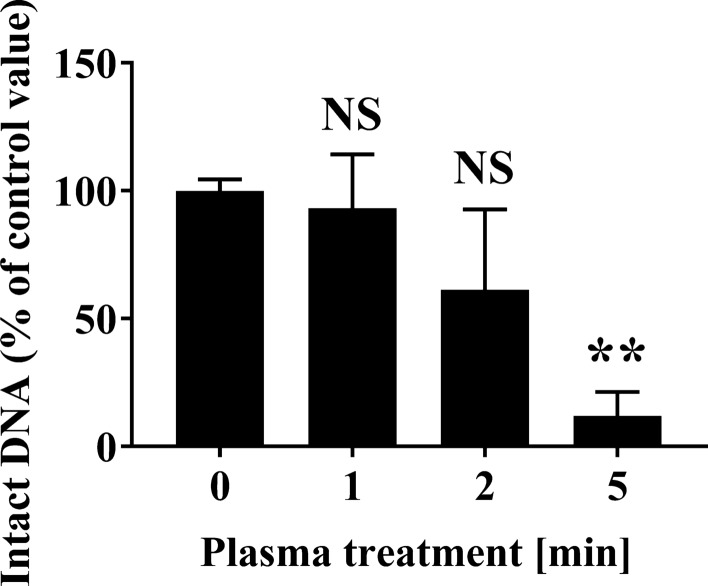
Changes to fungal DNA (28S rDNA) of *Penicillium venetum* spotted onto an aluminium plate following APDBD treatment. The level of intact 28S rDNA from *P. venetum* spotted onto an aluminium plate was quantified by real-time PCR. The level of the corresponding amplified product for the 0 min sample was assigned a value of 100%. Differences where *p* < 0.01(**) *versus* Control (0 min) were considered significant when verified by the non-repeated measured ANOVA followed by the Bonferroni correction. NS means no significant difference compared to Control (0 min)

**Fig. 4:**
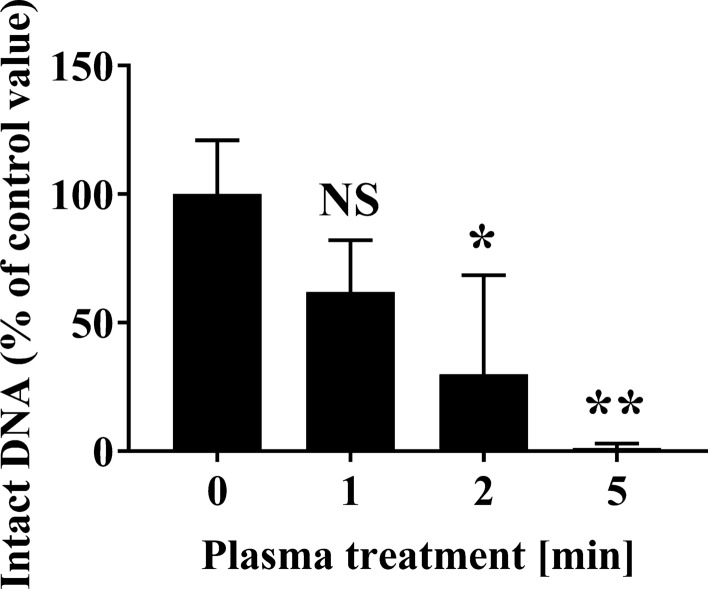
Changes to fungal DNA (28S rDNA) of *A. brasiliensis* spotted onto an aluminium plate following APDBD treatment. The level of intact 28S rDNA from *A. brasiliensis* spotted onto an aluminium plate was quantified by real-time PCR. The level of the corresponding amplified product for the 0 min sample was assigned a value of 100%. Differences where *p* < 0.05(*) and *p* < 0.01(**) *versus* Control (0 min) were considered significant when verified by the non-repeated measured ANOVA followed by the Bonferroni correction. NS means no significant difference compared to Control (0 min)

We analyzed the effect of plasma treatment on *Penicillium* and *Aspergillus* DNA by performing real-time PCR of the 28S rDNA D1/D2 region. The real-time PCR analysis of *P. venetum* 28S rDNA D1/D2 showed that the levels of intact DNA were 93.21 ± 12.10% at 1 min, 61.34 ± 18.11% at 2 min, but significantly decreased to 11.98 ± 5.39% at 5 min, when compared with the Control (0 min) (100.00 ± 2.54%) (Fig. 3). In addition, the results of the real-time PCR analysis of *A. brasiliensis* 28S rDNA D1/D2 showed the levels of intact DNA were 61.95 ± 11.65% at 1 min, but significantly decreased to 29.93 ± 22.20% at 2 min and 1.25 ± 0.97% at 5 min, when compared with the Control (0 min) (100.00 ± 12.08%) (Fig. 4).

### Decrease in the viable cell number of *P. venetum* spotted onto the pericarp of citrus fruits after APDBD treatment

**Fig. 5:**
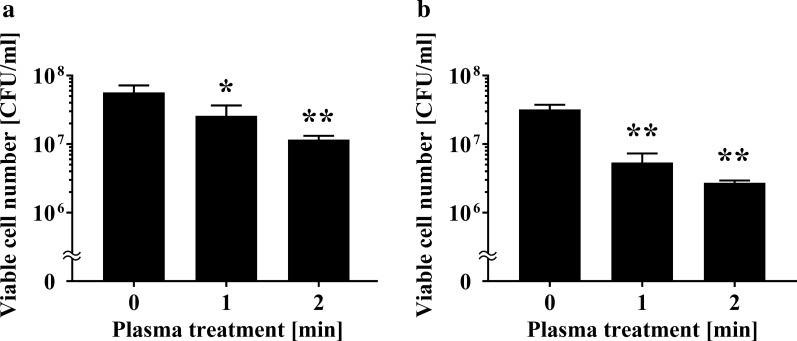
Viable cell number of *P. venetum* spotted onto the pericarp of citrus fruits following APDBD treatment. The viable cell number is expressed as CFU per ml. CFU/ml of *P. venetum* present on the contaminated pericarps of **a** ‘Kiyomi’ (*C. unshiu* × *C. sinensis*) and **b** ‘Kawano-natsudaidai’ (*C. natsudaidai*) at each individual APDBD treatment time (0, 1, or 2 min) are shown. Differences where *p* < 0.05(*) and *p* < 0.01(**) versus control (0 min) were considered significant when verified by the non-repeated measured ANOVA followed by the Bonferroni correction

Next, we investigated changes in the viable cell number of *P. venetum* spotted onto the pericarps of citrus fruits after APDBD treatment. A 20 μl aliquot of fungal suspension was spotted onto the pericarps of 'Kiyomi' (*Citrus unshiu* × *C. sinensis*) or ‘Kawano natsudaidai’ (*C. natsudaidai*) and air-dried. Samples were then subjected to APDBD treatment for 0, 1, or 2 min. The spots were subsequently collected with a sterilized cotton swab. The results of viable cell number on ‘Kiyomi’ was 5.67 × 10^7^ ± 1.20 × 10^7^ CFU/ml at 0 min, but significantly decreased to 2.60 × 10^7^ ± 0.61 × 10^7^ CFU/ml at 1 min and 1.17 × 10^7^ ± 0.09 × 10^7^ CFU/ml at 2 min (Fig. 5a). Viable cell number on 'Kawano natsudaidai' was 3.20 × 10^7^ ± 0.32 × 10^7^ CFU/ml at 0 min, but significantly decreased to 5.40 × 10^6^ ± 1.11 × 10^6^ CFU/ml at 1 min and 2.73 × 10^6^ ± 0.12 × 10^6^ CFU/ml at 2 min (Fig. 5b). From this data, the *D* value for ‘Kiyomi’ was 2.90 min, while that for ‘Kawano-natsudaidai’ was 1.88 min.

### Decrease in the viable cell number of *P. venetum* spotted onto the surface of citrus fruits after APDBD treatment

**Fig. 6:**
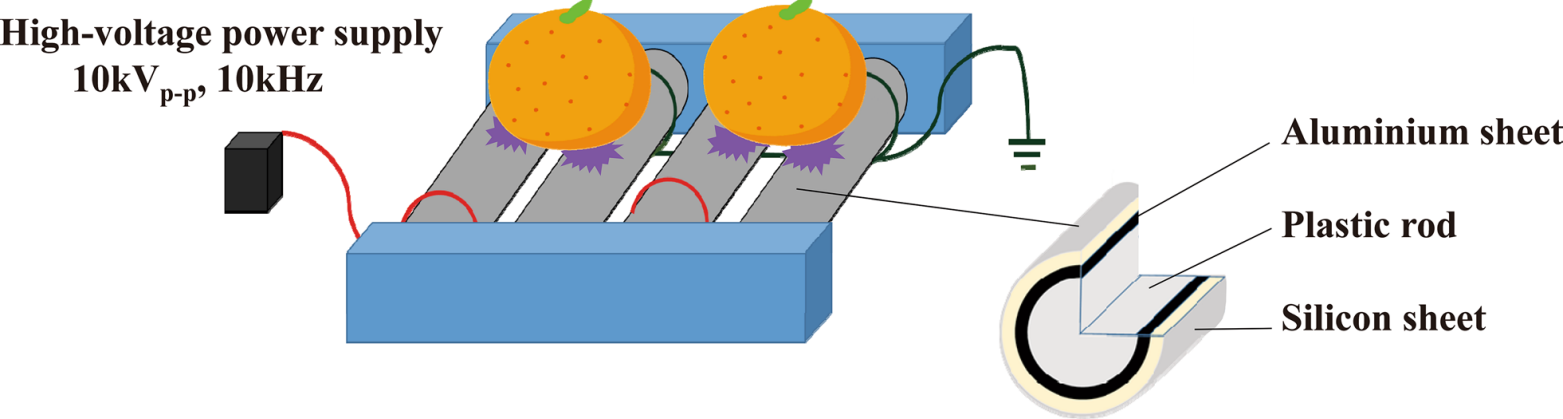
Schematic representation of atmospheric pressure dielectric barrier discharge (APDBD) treatment of the surface of citrus fruits. APDBD treatment was performed using a roller conveyor plasma instrument. The plasma device comprised high-voltage electrodes and earth electrodes, both of which were composed of a plastic rod covered with an aluminium sheet and silicon sheet (0.5 mm thick). A high-voltage power supply [10 kV_peak to peak_ (kV_p–p_), 10 kHz] was used for plasma generation. Plasma treatment of the citrus fruit was performed with both electrodes attached to a high-voltage electrode and an earth electrode

**Fig. 7:**
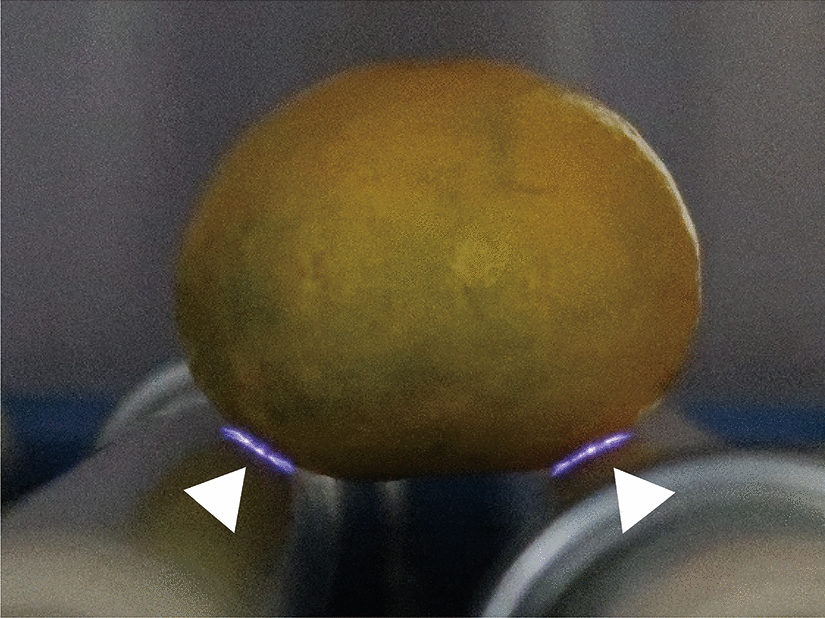
Emission observed during APDBD treatment of citrus fruit. An emission (purple light) is clearly visible between the surface of the electrodes and *C. unshiu* (arrowheads) during APDBD treatment using the roller conveyor plasma instrument

**Fig. 8:**
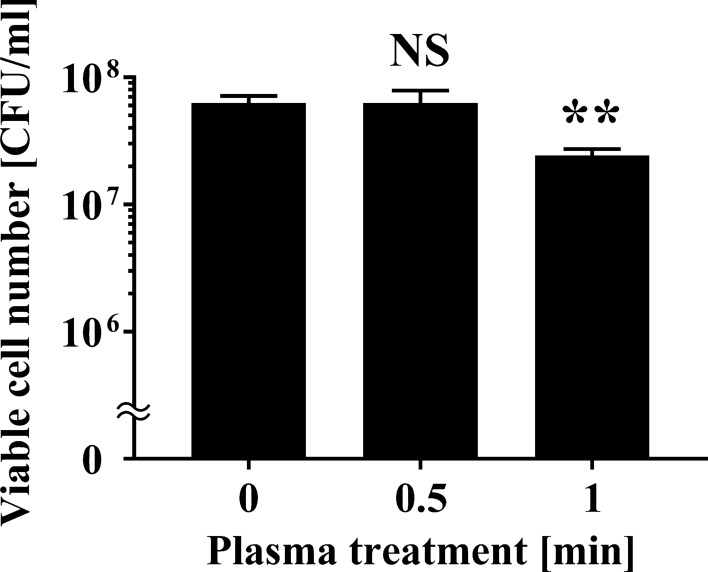
Viable cell number of *P. venetum* on the surface of *C. unshiu* following APDBD treatment. Viable cell numbers of *P. venetum*, expressed as CFU per ml, on the surface of contaminated *C. unshiu* at individual APDBD treatment times (0, 0.5, or 1 min) are shown. Differences where *p* < 0.01(**) versus control (0 min) were considered significant when verified by the non-repeated measured ANOVA followed by the Bonferroni correction. NS means no significant difference compared to Control (0 min)

Finally, we examined whether the roller conveyer plasma instrument was effective at disinfecting the surface of citrus fruits contaminated with fungus. A schematic representation of the roller conveyer plasma instrument is shown in Fig. 6. The light emitted during operation of the instrument is highlighted in Fig. 7. To investigate the disinfection effect of the plasma device, a 20 μl aliquot of fungal suspension was spotted onto the surface of *C. unshiu*, air-dried, and then plasma-treated for 0, 0.5, or 1 min. The spots were collected with a sterilized cotton swab. The results demonstrated that 1 min treatment with APDBD gave a significant disinfection effect for citrus fruit contaminated with *P. venetum* (Fig. 8). The results of viable cell number were 6.30 × 10^7^ ± 0.49 × 10^7^ CFU/ml at 0 min and unchanged at 6.30 × 10^7^ ± 0.90 × 10^7^ CFU/ml after 0.5 min treatment, but a significant decrease to 2.43 × 10^7^ ± 0.16 × 10^7^ CFU/ml after 1 min treatment. Based on this data, the *D* value was 2.42 min.

## Discussion

Recently, there has been significant interest in the potential antifungal application of plasma technology in the food industry. The antifungal effect of plasma on the food matrix has been studied using four different types of device; namely dielectric barrier discharge (DBD) plasma, plasma jet, corona-based plasma and microwave-powered plasma. *P. digitatum* on *C. unshiu* could be inactivated by DBD plasma (Yagyu et al. 2016). *A. flavus* on walnuts was also inactivated by a plasma jet (Amini and Ghoranneviss 2016). Corona-based plasma was shown to inactivate molds on cherry tomatoes (Lee et al. 2018) and broccoli seeds (Kim et al. 2017a). Microwave-powered plasma was effective at inactivating *A. brasiliensis* on onion powder (Kim et al. 2017b), and *A. flavus* on red peppers (Kim et al. 2014), as well as *P. italicum* on *C. unshiu* (Won et al. 2017).

Unfortunately, however, argon, nitrogen or helium are required as the inlet gas for operation of these devices, which makes them too costly to operate at scale (Moisan et al. 2001). Moreover, the sample box must be maintained at a low pressure, preventing the practical application of this new technology (Moisan et al. 2001). Recently, we developed a new type of roller conveyer instrument that generates plasma in an open system at atmospheric pressure. This device may provide a cost effective method for disinfecting agricultural products in a continuous process without using expensive inlet gases or having to maintain a low pressure environment. Our present results show that the roller conveyer instrument, which generates plasma on the basis of APDBD, can be used to efficiently disinfect fruit contaminated with *Penicillium* sp. (Figs. 1, 5 and 8) as well as *Aspergillus* sp. (Fig. 2).

Our experiments showed that plasma treatment of fungi-contaminated citrus fruits using the roller conveyer plasma instrument for 1 min resulted in a disinfection effect (Fig. 8). The *D* value was 2.90 min (‘Kiyomi’) and 1.88 min (‘Kawano-natsudaidai’) for APDBD treatment of fungal infection on the pericarps of the fruit (Fig. 5), and 2.42 min for APDBD-treatment of the fruit surface (Fig. 8). Thus, the disinfection efficiency of APDBD treatment is far superior to that of *P. digitatum* by UV-C treatment (*D* value = 26.2 min) (Iseki et al. 2010). These findings suggest that the roller conveyor type plasma instrument is useful for the surface disinfection of citrus fruits contaminated with fungi. In addition, APDBD treatment causes damage to the DNA of *Penicillium* as well as *Aspergillus*, because in both cases the plasma treatment results in decreased levels in intact DNA (Figs. 3 and 4). These observations suggest that DNA damage may, at least in part, contribute to the inactivation mechanism of APDBD treatment. Interestingly, the *D* value was significantly longer for plasma treatment of *Penicillium* spotted on the pericarp or surface of a citrus fruit compared with *Penicillium* spotted onto an aluminium plate. In addition, two classes of citrus pericarps showed different *D* values. Thus, the disinfection efficiency might depend on the surface material and agricultural products on which the fungi are located. However, the present study used a limited number of treatment conditions with only two classes of agricultural products and a control material. Moreover, slight variations in the initial fungal cell number, arising from lot-to-lot variation in fungal culture preparation, may have occurred. Thus, further investigation into the disinfection effect using a broad range of agricultural products and surface materials together with the corresponding effective treatment time at a variety of initial fungal cell numbers is required to confirm the difference of *D* value between abiotic and biotic surfaces as well as among agricultural products.

The mechanisms by which the instrument disinfects fungi remains unclear. Plasma can be produced by introduction of a high electric field to gases. The resultant partially ionized gases form plasma, which contain dissociated molecules, positive and negative ions, photons, and electrons, as well as reactive chemical species (Shintani et al. 2010). In our previous study using a roller conveyer plasma instrument, we employed optical emission spectroscopy and chemical indicators to detect reactive oxygen and nitrogen species (RONS) such as peroxynitrite (ONOO^−^) and hydrogen peroxide (H_2_O_2_) during operation of the instrument (Toyokawa et al. 2017). Several reports have shown that reactive chemical products generated by plasma disinfection techniques appears to contribute to inactivating fungi (Los et al. 2020). This hypothesis is supported by our results, which show that plasma generated by a roller conveyer plasma instrument induces changes in the fungal DNA (Figs. 3 and 4). Similarly, treatment with other plasma instruments that generate nitrogen gas plasma (BLP-TES) result in oxidation of genomic DNA mediated by reactive chemicals species (Sakudo et al. 2017a).

The present system using a roller conveyer plasma instrument can be applied to agricultural crops after harvesting to control infection during the sorting process. However, it is not known whether this treatment has a deleterious effect on the various agricultural products. Further investigation is required before practical application of this new technology can proceed. In addition, fungi produce metabolites, including mycotoxins that display carcinogenic and mutagenic activity (Sakudo et al. 2017b; Pankaj et al. 2018). It is possible that operation of the roller conveyor plasma instrument might inactivate these mycotoxins.

In conclusion, our study suggests that the novel roller conveyer plasma system can efficiently disinfect the surface of citrus fruits contaminated with fungi. We believe the roller conveyer plasma device could be used to disinfect citrus fruits after harvesting during the sorting process.

## Supplementary Information

**Additional file 1: Figure S1.** Voltage waveforms during operation of the roller conveyer plasma instrument.

## Data Availability

All the relevant data used to support the findings of this study are included within the article.
