# Supplementary material for: Application of a roller conveyor type plasma disinfection device with fungus-contaminated citrus fruits
Source: AMB Express. 2021 Jan 9;11:16. doi: 10.1186/s13568-020-01177-2 (PMC7797019; doi:10.1186/s13568-020-01177-2)
Supplement: Supplementary file 1 — Additional file 1: Figure S1. Voltage waveforms during operation of the roller conveyer plasma instrument. [file 13568_2020_1177_MOESM1_ESM.docx]

**Additional file**

Application of a roller conveyor type plasma disinfection device with fungus-contaminated citrus fruits

Akikazu Sakudo^1,2^* and Yoshihito Yagyu^3^

^1^ School of Veterinary Medicine, Okayama University of Science, Imabari, Ehime, Japan; akikazusakudo@gmail.com

^2^ Laboratory of Biometabolic Chemistry, School of Health Sciences, University of the Ryukyus, Nishihara, Okinawa, Japan

^3^ Department of Electrical and Electric Engineering, Sasebo National College of Technology, Sasebo, Nagasaki 857-1193, Japan

***** Correspondence: akikazusakudo@gmail.com

**Figure S1 Voltage waveforms during operation of the roller conveyer plasma instrument**

Voltage waveforms in **a** steady condition (without any object placed on the electrodes) or object treatment condition (either **b** aluminium plate or **c** citrus fruit) measured using an oscilloscope (DS-5554; Iwatsu Electric Co., Ltd., Tokyo, Japan) with a high-voltage probe (P6015A; Tektronix, Inc., Beaverton, OR).
